# Nanocrystalline TiO_2_/SnO_2_ heterostructures for gas sensing

**DOI:** 10.3762/bjnano.8.12

**Published:** 2017-01-12

**Authors:** Barbara Lyson-Sypien, Anna Kusior, Mieczylaw Rekas, Jan Zukrowski, Marta Gajewska, Katarzyna Michalow-Mauke, Thomas Graule, Marta Radecka, Katarzyna Zakrzewska

**Affiliations:** 1 AGH University of Science and Technology, Faculty of Computer Science, Electronics and Telecommunications, Al. A. Mickiewicza 30, 30-059 Krakow, Poland; 2 AGH University of Science and Technology, Faculty of Materials Science and Ceramics, Al. A. Mickiewicza 30, 30-059 Krakow, Poland; 3 AGH University of Science and Technology, Academic Center for Materials and Nanotechnology, Al. A. Mickiewicza 30, 30-059 Krakow, Poland; 4 EMPA, Swiss Federal Laboratories for Materials Testing and Research, Laboratory for High Performance Ceramics, Uberlandstrasse 129, 8600 Duebendorf, Switzerland; 5 Paul Scherrer Institut, 5232 Villigen PSI, Switzerland

**Keywords:** gas sensors, hydrogen, n–n heterojunctions, nanomaterials, TiO_2_/SnO_2_

## Abstract

The aim of this research is to study the role of nanocrystalline TiO_2_/SnO_2_ n–n heterojunctions for hydrogen sensing. Nanopowders of pure SnO_2_, 90 mol % SnO_2_/10 mol % TiO_2_, 10 mol % SnO_2_/90 mol % TiO_2_ and pure TiO_2_ have been obtained using flame spray synthesis (FSS). The samples have been characterized by BET, XRD, SEM, HR-TEM, Mössbauer effect and impedance spectroscopy. Gas-sensing experiments have been performed for H_2_ concentrations of 1–3000 ppm at 200–400 °C. The nanomaterials are well-crystallized, anatase TiO_2_, rutile TiO_2_ and cassiterite SnO_2_ polymorphic forms are present depending on the chemical composition of the powders. The crystallite sizes from XRD peak analysis are within the range of 3–27 nm. Tin exhibits only the oxidation state 4+. The H_2_ detection threshold for the studied TiO_2_/SnO_2_ heterostructures is lower than 1 ppm especially in the case of SnO_2_-rich samples. The recovery time of SnO_2_-based heterostructures, despite their large responses over the whole measuring range, is much longer than that of TiO_2_-rich samples at higher H_2_ flows. TiO_2_/SnO_2_ heterostructures can be intentionally modified for the improved H_2_ detection within both the small (1–50 ppm) and the large (50–3000 ppm) concentration range. The temperature *T*_max_ at which the semiconducting behavior begins to prevail upon water desorption/oxygen adsorption depends on the TiO_2_/SnO_2_ composition. The electrical resistance of sensing materials exhibits a power-law dependence on the H_2_ partial pressure. This allows us to draw a conclusion about the first step in the gas sensing mechanism related to the adsorption of oxygen ions at the surface of nanomaterials.

## Introduction

The TiO_2_–SnO_2_ system is extremely important for gas sensing as already proved by many works already published [1–12]. Particularly interesting is the generally accepted possibility of two scenarios: the formation of solid solutions within a certain compositional and temperature range [1,3] or composites of TiO_2_ and SnO_2_ [4–7]. The fundamental and up till now unresolved question is which form is better for gas sensing.

In fact, we have found in our research that more than two cases are possible. Our previous experience with this system [1,4,6–7,13] indicates that four classes of materials can be obtained:

A. a simple mixture of the constituents denoted as TiO_2_–SnO_2_

B. perfect solid solutions Ti*_x_*Sn_1−_*_x_*O_2_ where 0 ≤ *x* ≤1

C. partially decomposed Ti*_x_*Sn_1−_*_x_*O_2_–Sn*_y_*Ti_1−_*_y_*O_2_ where 0 ≤ *x* ≤ 1 and 0 ≤ *y* ≤ 1

D. decorated nano-heterostructures denoted as TiO_2_@SnO_2_, e.g., TiO_2_ nanoflowers overcoated with SnO_2_ nanoparticles

Synergetic effects and catalytic reactions can be expected in the case of A) and C) while changes of the morphological and the electronic structure dominate in the case of B) and D). Surface phenomena determine the gas-sensor response in the case of decorated nano-heterostructures, D). As shown in [6], electron transfer over n–n-type heterojunctions can account for sensor sensitization in the cases of A) and D). The formation of n–n-type heterojunctions at the contact between SnO_2_ and TiO_2_ grains and its effect on the enhancement of the sensor response has been reviewed recently [14].

**Figure 1:**
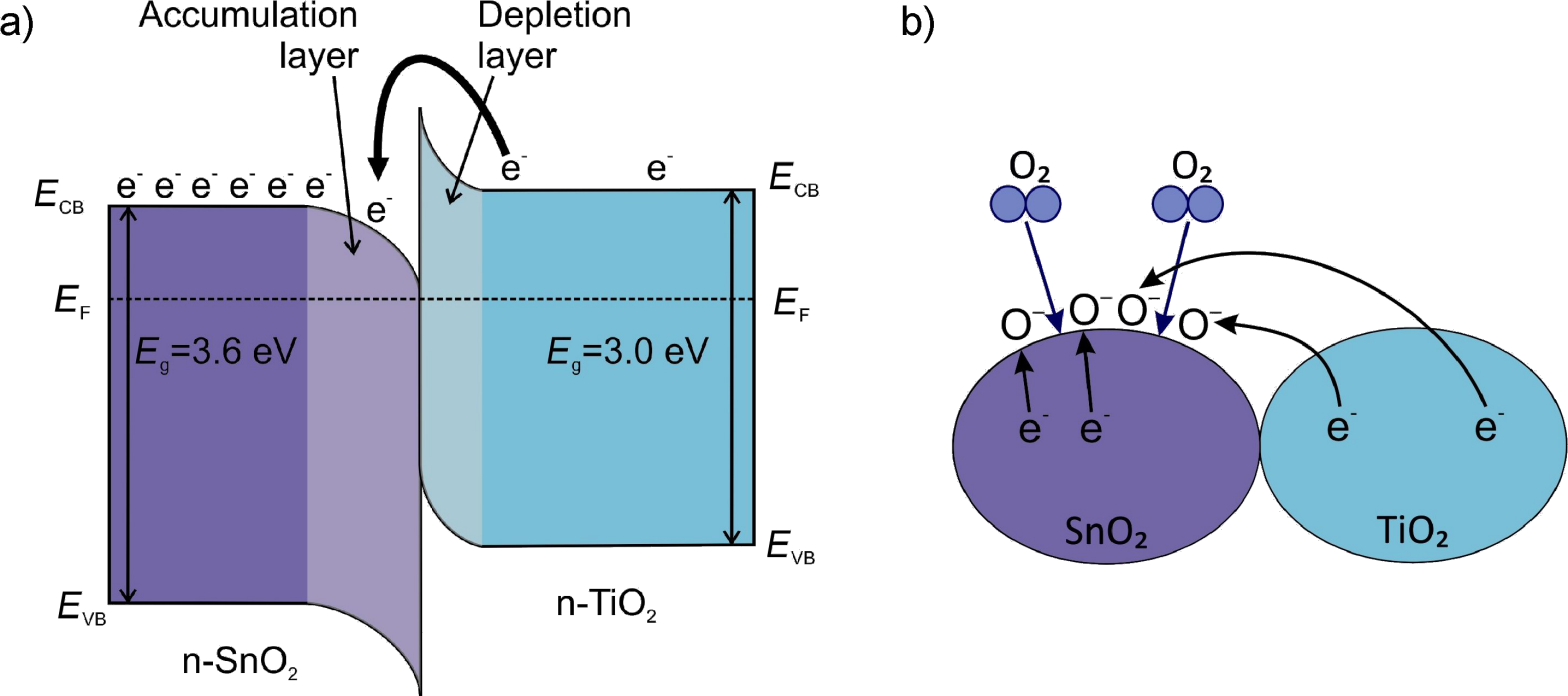
Schematics illustrating the beneficial action of n–n heterojunctions for the sensitization of the gas sensor. (a) Electronic band diagram of an n–n heterojunction, b) electron transfer from a TiO_2_ to a SnO_2_ grain providing active gas adsorption sites. *E*_F_: Fermi energy, E_VB_: valence band maximum energy, E_CB_: conduction band minimum energy, *E*_g_: energy band gap, e^−^: electron, O^−^: singly ionized oxygen adatom.

Figure 1 explains why the formation of n–n heterojunctions between TiO_2_ and SnO_2_ grains enhances the response of the gas sensor. In fact, the sensitization comes to an effect in the first step of reducing gas detection, namely the preadsorption of oxygen at the grain surface (in our case it is assumed to be in the form of O^−^ as shown in Figure 1b). The efficiency of the O^−^ adsorption process is greatly enhanced when a sufficient concentration of electrons is provided. It is usually assumed that SnO_2_ grains are more suitable for oxygen adsorption, thus electron transfer from TiO_2_ grains is necessary to increase the number of adsorption sites. Electron transfer from TiO_2_ to SnO_2_ is provided by an appropriate electronic configuration because both conduction (CB) and valence (VB) band edges of TiO_2_ are above those of SnO_2_ as shown in Figure 1a. The potential difference that is formed when TiO_2_ and SnO_2_ grains come to contact facilitates electron transport from TiO_2_ to SnO_2_ thus promoting oxygen preadsorption at the surface of SnO_2_ grains.

In the publications about the TiO_2_–SnO_2_ system [1–12,15–17] for improved gas sensing, one can find different types of materials: thick and thin films, nanostructures such as nanofibers, nanorods, nanobelts, nanopowders as well as core–shell particles. Table 1 presents some of the examples of the latest papers dealing with TiO_2_–SnO_2_ materials for the detection of different gases.

**Table 1:** TiO_2_–SnO_2_ systems for gas sensing. The best response is defined either as: *R*_0_/*R* (for *n*-type material + reducing gas and *p*-type material + oxidizing gas) or *R*/*R*_0_ (for *n*-type material + oxidizing gas and *p*-type material + reducing gas), where *R*_0_ denotes the electrical resistance in the reference atmosphere and *R* is the electrical resistance under exposure to the detected gas.

Form	Method of preparation	Composition/characteristic features	Experimental conditions	Best response	Ref. year

nanopowders	co-precipitation of SnO_2_ and TiO_2_, mechanical mixing	wt % of SnO_2_: 100, 90, 70, 0	H_2_ (1–500 ppm) 300–500 °C	*R*_0_/*R* = 12; 70 wt % SnO_2_ + 30 wt % TiO_2_; 20 ppm H_2_; 400 °C	[2] 2012
	commercial Sigma Aldrich SnO_2_ and TiO_2_, mechanical mixing	mol % of SnO_2_: 100, 95, 90, 80, 50, 20, 10, 2, 0	H_2_ (50–3000 ppm) 250–400 °C	*R*_0_/*R* ≈ 90; 50 mol % SnO_2_ + 50 mol % TiO_2_; 500 ppm H_2_; 325 °C	[4,6] 2013
			NH_3_ (100–5000 ppm) 400 °C	*R*/*R*_0_ = 1.49; 50 mol % SnO_2_ + 50 mol % TiO_2_; 1200 ppm NH_3_; 400 °C	[7] 2012
	sol–gel SnO_2_ and TiO_2_, mechanical mixing	Ti/Sn: 0, 0.1, 0.3, 0.5, 0.8	VOCs (50–400 ppm) 200–450 °C	*R*_0_/*R* = 65 Ti/Sn: 0.1; 200 ppm VOCs; ca. 350 °C	[5] 2010
	symplectic gel co-precipitation (SGC)	Ti*_x_*Sn_1−_*_x_*O_2_ (*x*: 0.3, 0.5, 0.7, 0.9)	CO, CH_4_, NO_2_ (50 ppm) 450–650 °C	*R*_0_/*R* = 7; Ti_0.3_Sn_0.7_O_2_; 50 ppm CO; 500 °C	[3] 2009
nanofibers	commercial SnO_2_ and TiO_2_	mol % TiO_2_: 100, 90	H_2_ (5000–20000 ppm) 300–600 °C	*R*_0_/*R* ≈ 1.25; TiO_2_; 5000 ppm H_2_; 500 °C	[8] 2005
polycrystalline ceramics, thin films	solid-state reactions, rf reactive sputtering	Sn_1−_*_x_*Ti*_x_*O_2_ where *x*: 0, 0.05, 0.1, 0.9, 0.95, 1	H_2_ (330–20000 ppm) 400–650 °C	*R*/*R*_0_ = 2.63; TiO_2_; 1000 ppm H_2_; 550 °C	[1] 1998
thin films	plasma-enhanced atomic layer deposition	SnO_2_ thin films grown on TiO_2_ single crystals	H_2_, NH_3_, CO (100–1000 ppm) 300–500 °C	*R*_0_/*R* ≈ 380; (101)SnO_2_ on (101) TiO_2_; 1000 ppm H_2_; 400 °C	[9] 2010
thick film	sol–gel	Ti/Sn: 1/7	VOCs (200 ppm) 200–400 °C	*R*_0_/*R* ≈ 55; Ti/Sn: 1/7; 200 ppm VOCs; 280–360 °C	[10] 2010
coral-like nanostructures	hydrothermal method	coral-like SnO_2_ nanostructures modified with TiO_2_ nanoparticles	VOCs (50–200 ppm) 200 °C	*R*_0_/*R* ≈ 12; 200 ppm benzene; 200 °C	[11] 2012
nanorods	thermal evaporation and metal-organic chemical vapor deposition	SnO_2_ nanorods with TiO_2_ capping	NO_2_ (50–100 ppm) 100 °C	*R*/*R*_0_ = 2.85; 50 ppm NO_2_; 100 °C	[15] 2012
nanobelts	hydrothermal method	SnO_2_ nanoparticles deposited on TiO_2_ nanobelts	VOCs (10–500 ppm) 160–410 °C	*R*_0_/*R* ≈ 50; 500 ppm acetone; 350 °C	[12] 2015
core–shell	sol–gel	nanocomposite Ti/Sn: 1/1, 1/1.5, 1/2	ethanol (500-5000 ppm) 140-420^o^C	*R*_0_/*R* = 70; Ti/Sn: 1/1.5; 5000 ppm ethanol; 220^o^C	[16] 2012
	single-needle electrospinning	hollow SnO_2_ nanofibers and core–shell TiO_2_–SnO_2_ nanofibers	VOCs (10–1000 ppm) 200–370 °C	*R*_0_/*R* = 55; 1000 ppm ethanol; 300 °C	[17] 2016

The performance of a resistive-type gas sensor is inherently related to the form and number of oxygen species adsorbed at the surface of the sensing material in the first step [18]. The equation describing the oxygen chemisorption can be written as [19]:

[1]
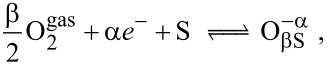


where 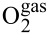 is an oxygen molecule in the ambient atmosphere, *e*^−^ is an electron that can reach the surface, S is an unoccupied chemisorption site, and 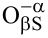 represents chemisorbed oxygen species with α = 1 for singly ionized forms, α = 2 for doubly ionized forms, β = 1 for atomic forms and β = 2 for molecular oxygen.

Table 2 presents possible oxygen species that can be chemisorbed at the surface of the gas sensing material.

**Table 2:** Possible combinations of α and β and the resulting chemisorbed oxygen species.

	α	β	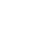

molecular	1	2	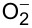
atomic	1	1	O^−^
atomic	2	1	O^2−^

Hydrogen is considered to react in a second step, at the surface of the oxides, with preadsorbed or lattice oxygen, which, in consequence, increases the electronic conduction. The surface reaction between hydrogen and oxygen can be described in general by the following equation:

[2]
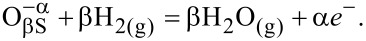


It has been observed that as the result of the two step interaction described above, the electrical resistance, *R*, of the sensor for any reducing gas can be expressed as [1,20]:

[3]
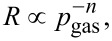


where *p*_gas_ is the partial pressure of the reducing gas while the power coefficient *n* is specific to the kind of the target gas and particular reaction with oxygen species preadsorbed at the surface of the semiconductor.

In our previous work [1] one can find an analysis of TiO_2_–SnO_2_ in the form of polycrystalline ceramics and rf-sputtered thin films upon interaction with H_2_ but not much is known in the case of nanomaterials. Since that time we have focused on commercial TiO_2_ and SnO_2_ starting nanomaterials for the sensing of H_2_ and NH_3_ [4,6–7]. One of the main conclusion from our latest research on commercial materials is that a small addition of TiO_2_ to SnO_2_ affects gas sensing characteristics to a large extent [4,6]. In this work, for the first time**,** we intend to demonstrate nano-heterostructures of the TiO_2_–SnO_2_ system prepared by flame spray synthesis with application to gas sensing.

Flame spray synthesis is a well-known and efficient method for the synthesis of crystallized metal oxide nanopowders with particular morphology, e.g., spherical, monodispersed nanoparticles of TiO_2_ with good photocatalytic properties [21–23]. However, its application to nano-heterostructures for gas sensing is not known.

The aim of the current work is to study the role of nanocrystalline TiO_2_/SnO_2_ n–n heterojunctions for hydrogen sensing. Within this work the detailed study on crystallographic structure, morphology, electrical properties, H_2_ sensing behavior and the power-law nature of the electrical resistance of TiO_2_/SnO_2_ heterostructures is presented. The influence of water adsorption and desorption on the electrical properties of TiO_2_/SnO_2_ is also taken into account. The detection threshold is studied for the first time as a function of the chemical composition of TiO_2_/SnO_2_ heterostructures.

## Experimental

Nanopowders of the TiO_2_–SnO_2_ system were obtained by flame spray synthesis, FSS, technique using an oxygen–acetylene flame. Not only end compositions, 100 mol % SnO_2_ and 100 mol % TiO_2_ were prepared. Potential heterostructures of 90 mol % SnO_2_/10 mol % TiO_2_ and 10 mol % SnO_2_/90 mol % TiO_2_ were synthesized as well.

Titanium diisopropoxide bis(acetylacetonate) (TiC_16_H_28_O, 75 wt % in isopropanol, ABCR, CAS: 17927-72-9) and tetramethyltin (CAS: 594-27-4), dissolved in absolute ethanol (C_2_H_5_OH, 99%, Sigma Aldrich) were used as precursors of titanium and tin, respectively. Details of the FSS setup have been reported elsewhere [24–25].

The required composition and specific surface area (SSA) were obtained by adjusting the ratio of the precursors in the precursor mixture, the total flow rate of which was kept constant at 12.64 cm^3^·min^−1^. The total precursor concentration in the flame was kept constant at 1.5 mol·kg^−1^. The precursor solution was fed by a syringe pump and was atomized with oxygen (583 cm^3^·s^−1^) in a gas-assisted external mixing nozzle. The combustible aerosol was ignited by six oxygen-acetylene flamelets (C_2_H_2_, 217 cm^3^·s^−1^; O_2_, 283 cm^3^·s^−1^) and the produced particles were collected on glass-fiber filters (GF/A 150, Whatman) using vacuum pumps. The nanopowders of TiO_2_–SnO_2_ did not require any post-synthesis heat treatment since the technique provides well crystallized nanostructures.

The specific surface area (SSA) was determined using Brunauer–Emmett–Teller (BET) nitrogen-adsorption isotherms obtained with a Beckman–Coulter SA3100 apparatus.

The crystallographic structure was analyzed on the basis of XRD patterns recorded in Bragg–Brentano configuration with the help of a Philips X’Pert Pro diffractometer. Based on Rietveld refinement it was possible to determine the weight fractions of cassiterite SnO_2_, rutile TiO_2_ and anatase TiO_2_, the lattice constants and the crystallite sizes, *d*_XRD_.

The ^119^Sn Mössbauer effect measurements were performed in transmission geometry using an MS-4 RENON spectrometer and CaSnO_3_ as source. The Mössbauer spectra were fitted using a transmission integral in order to take into account the absorber thickness effects. The spectra were refined with quadrupole doublets of Lorentzian lines assuming a non-zero value of the electric field gradient at the tin site. Hyperfine parameters, the isomer shift, *IS*, and quadrupole splitting, *QS*, as well as the full width at half maximum of the Sn peaks, *G*, were found. The values of isomer shift are given relative to the CaSnO_3_ source kept at room temperature.

Morphology of the synthesized TiO_2_–SnO_2_ nanomaterials was studied by means of scanning electron microscopy (SEM) performed with a FEI Nova Nano SEM 200 apparatus. High-resolution transmission electron microscopy (HR-TEM) images were obtained using a FEI Tecnai TF 20 X-TWIN microscope. Mapping of chemical elements and diffraction patterns were provided.

The electrical properties were investigated by impedance spectroscopy (IS) in the temperature range from 20 to 550 °C in air. The impedance spectroscopy measurements were performed with a Solatron system (Fra 1260 + dielectric interface 1294). Experimental parameters and data acquisitions were controlled with the FRA software. A frequency range from 1 to 10^6^ Hz was covered, with 10 mV amplitude. The impedance spectra were analyzed using the ZView software. An equivalent circuit containing one resistor and a constant phase element (CPE) was used for fitting.

In order to perform gas sensing measurements, the nanosensors were prepared in the form of tablets that were pressed from powders under a pressure of 25 MPa, then annealed at 400 °C and covered with planar silver electrodes. The detailed description of the experimental setup used for the H_2_-sensing measurements can be found in [26–27]. The desired hydrogen concentration was obtained by using mass flowmeters mixing synthetic air (reference gas) with H_2_ (0.01% H_2_, 0.1% H_2_, 1% H_2_ + Ar depending on the concentration range, i.e., 1–30 ppm, 5–300 ppm and 50–3000 ppm H_2_, respectively). The total gas (hydrogen mixture + air) flow rate was kept constant at 120 sccm. The measurements were carried out in dry atmosphere. The synthetic air contained less than 1 ppm of water vapor while that of hydrogen + argon mixture had less than 10 ppm of contaminants. The relative humidity level was verified to be of about 0–1% RH at room temperature.

Dynamic changes in the electrical resistance upon hydrogen exposure have been detected over a low-to-medium concentration range of 1–3000 ppm at a constant temperature between 200 and 400 °C. Measurements within an interval of 1–50 ppm H_2_ were performed to determine the hydrogen detection limit. The sensor response *S* was defined as the ratio between the electrical resistance in the reference atmosphere, *R*_0_, and the electrical resistance upon interaction with hydrogen, *R*:

[4]
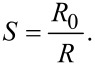


## Results and Discussion

**Figure 2:**
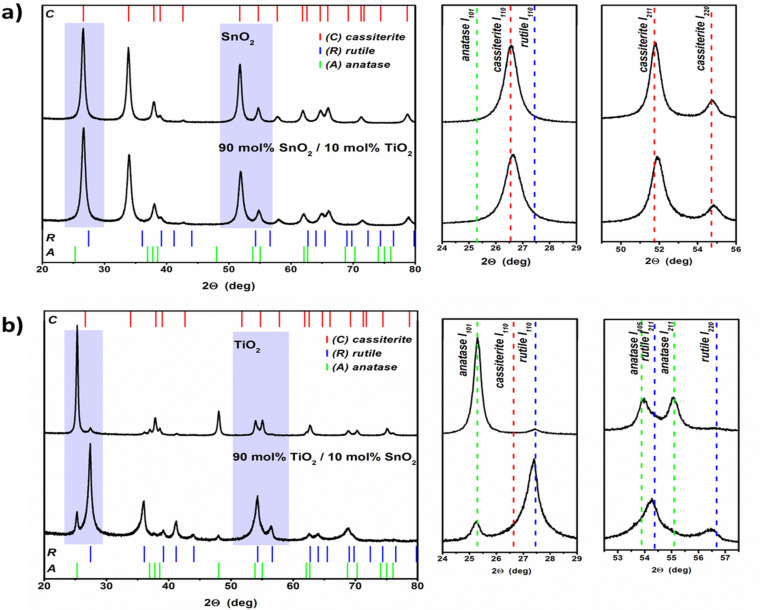
Comparison between XRD patterns of a) SnO_2_ and 90 mol % SnO_2_/10 mol % TiO_2_; b) TiO_2_ and 90 mol % TiO_2_/10 mol % SnO_2_ nanopowders.

Figure 2 shows XRD patterns for SnO_2_-rich (Figure 2a) and TiO_2_-rich (Figure 2b) heterostructured nanopowders obtained by FSS compared with the pure end components SnO_2_ and TiO_2_. Table 3 recapitulates the results of XRD Rietveld refinement performed for TiO_2_/SnO_2_ nanopowders, and the SSA values determined from BET measurements. The FSS parameters were chosen intentionally in order to obtain approximately the same SSA, and according to the expectation the SSA were found to be within a range of 54–62 m^2^·g^−1^, independent of the chemical composition.

**Table 3:** The results of BET and XRD Rietveld refinement of TiO_2_–SnO_2_ nanomaterials; “A” denotes anatase, “R” rutile and “C” cassiterite; SSA: specific surface area.

Sample	XRD	C SnO_2_	A TiO_2_	R TiO_2_	SSA (m^2^/g)

SnO_2_	wt %	100			54
	*a* (nm) *c* (nm)	0.47360 0.31857			
	crystallite size (nm)	12			
10 mol % TiO_2_/90 mol % SnO_2_	wt %	100			62
	*a* (nm) *c* (nm)	0.47299 0.31780			
	crystallite size (nm)	10			
90 mol % TiO_2_/10 mol % SnO_2_	wt %	18.4	8.5	73.1	60
	*a* (nm) *c* (nm)	0.45956 0.30447	0.37840 0.95489	0.46007 0.29664	
	crystallite size (nm)	3	27	14	
TiO_2_	wt %		91.1	8.9	57
	*a* (nm) *c* (nm)		0.37849 0.94997	0.45911 0.29440	
	crystallite size (nm)		25	15	

As it can be concluded from Table 3 and Figure 2, pure SnO_2_ exhibits the crystallographic structure of cassiterite, whereas in the case of pure TiO_2_ two polymorphic forms, anatase and rutile, are present with a predominance of anatase (91.1 wt %). Lattice constants *a* and *c* of both TiO_2_ and SnO_2_ determined experimentally are in good agreement with their theoretical values [28], TiO_2_ rutile: *a* = 0.45911 nm, *c* = 0.29440 nm; TiO_2_ anatase: *a* = 0.37849 nm, *c* = 0.94997 nm; SnO_2_ cassiterite: *a* = 0.47360 nm, *c* = 0.31857 nm.

The main conclusion from Figure 2a is that one can observe a systematic shift of all cassiterite SnO_2_ XRD peaks towards higher diffraction angles resulting from a decrease in the lattice constants *a* and *c* (Table 3) of the 90 mol % SnO_2_/10 mol % TiO_2_ nanopowder compared with 100% SnO_2_. This effect is typical and is usually interpreted as Ti substitution at Sn lattice sites [1]. The absence of non-identified peaks belonging to TiO_2_ phases supports the conclusion that under these conditions a solid solution is formed (case B). However, the presence of heterojunctions between the small amount of TiO_2_ grains well dispersed within the primary SnO_2_ cassiterite phase cannot be excluded.

The influence of 10 mol % SnO_2_ in TiO_2_ on the XRD pattern (Figure 2b) is much more pronounced. As observed previously for polycrystalline ceramics and thin films [1], even a relatively small amount of SnO_2_ in TiO_2_ results in a dramatic reconstruction of the crystallographic structure. The content of anatase decreases from 91.1 wt % for pure TiO_2_ to 8.5 wt % for 10 mol % SnO_2_/90 mol % TiO_2_. The crystallite size of anatase remains unchanged (25 nm for 100% TiO_2_ and 27 nm for 10 mol % SnO_2_/90 mol % TiO_2_). The rutile TiO_2_ phase begins to predominate (71.1 wt %) with lattice constants slightly higher than those of 100% TiO_2_ indicating partially decomposed solid solution (case C). Furthermore, the evidence of precipitation of 18.4 wt % SnO_2_ cassiterite with very small crystallites of about 3 nm favors the hypothesis of a heterostructure formation.

**Figure 3:**
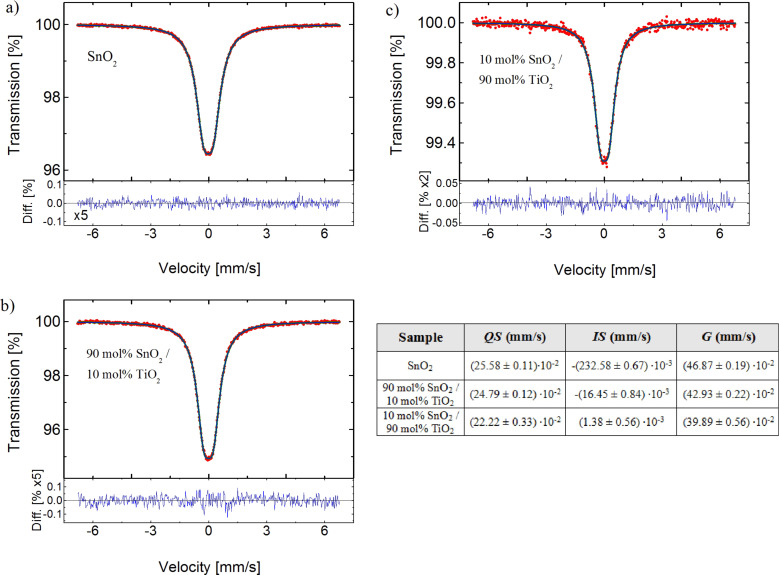
Mössbauer transmission spectra of: a) SnO_2_; b) 90 mol % SnO_2_/10 mol % TiO_2_; c) 90 mol % TiO_2_/10 mol % SnO_2_ nanopowders. *IS* denotes the isomer shift, *QS* is the quadrupole splitting, whereas *G* represents the full width at half maximum.

In order to study the possible tin oxidation states, Mössbauer spectroscopy was applied. Figure 3 demonstrates transmission spectra of: a) SnO_2_; b) 90 mol % SnO_2_/10 mol % TiO_2_ and c) 90 mol % TiO_2_/10 mol % SnO_2_ nanopowders.

The observed peaks are characteristic for Sn^4+^ (SnO_2_) for all presented powder samples. No contribution from Sn^2+^ (SnO) has been detected. The measured hyperfine parameters of SnO_2_**,** i.e.**,** the isomer shift, *IS*, and quadrupole splitting, *QS*, at room temperature exhibit similar values for all the studied samples and are in agreement with those reported for SnO_2_ [29–31].

**Figure 4:**
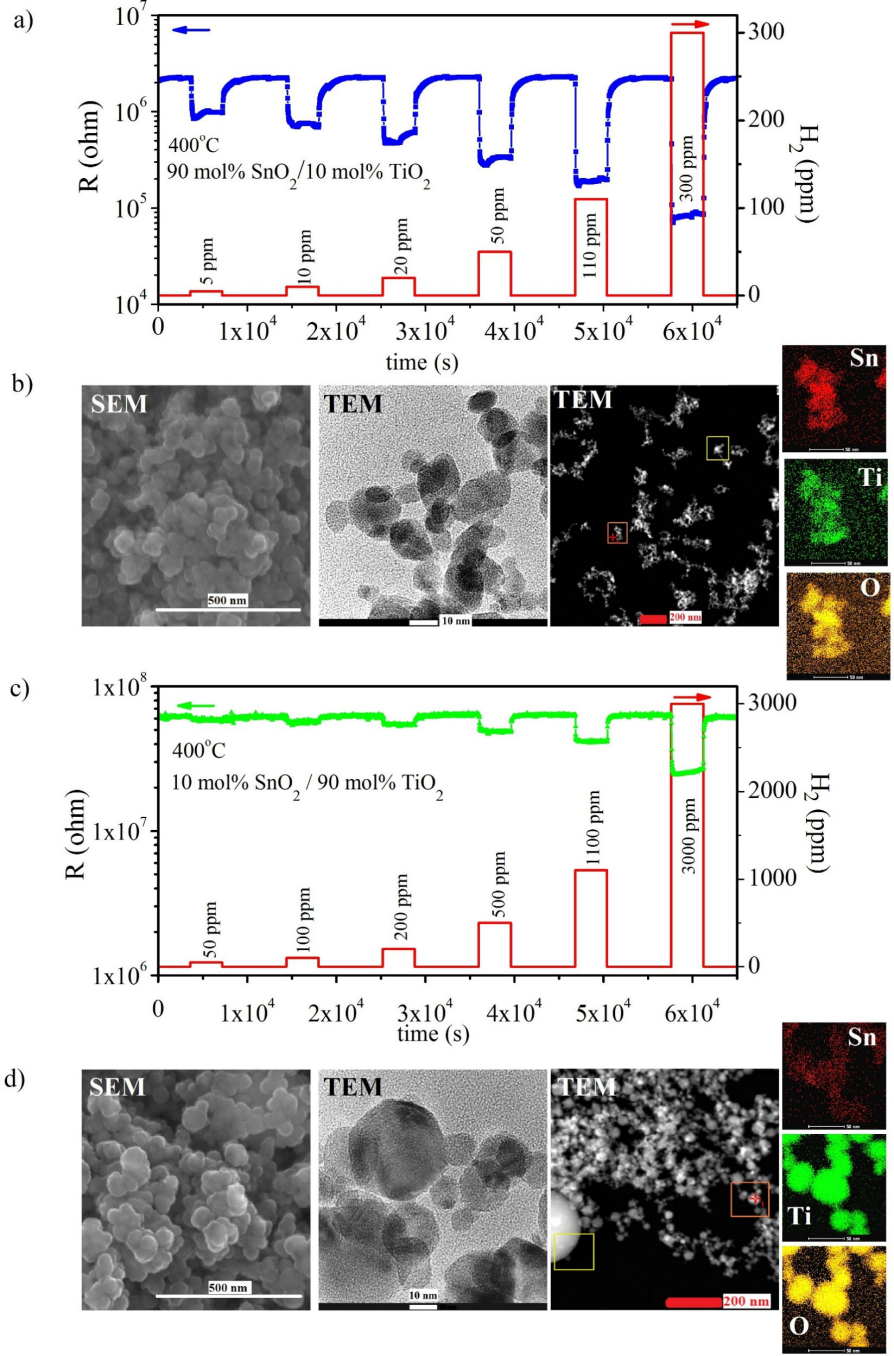
Dynamic changes in the electrical resistance, *R*, of: a) 90 mol % SnO_2_/10 mol % TiO_2_ (H_2_ concentration: 5–300 ppm) and c) 10 mol % SnO_2_/90 mol % TiO_2_ (H_2_ concentration: 50–3000 ppm) nanomaterials upon interaction with hydrogen at a constant temperature of 400 °C along with the corresponding SEM and TEM images (b, d). Step changes in hydrogen concentrations are given on the right hand scale (a, c).

Figure 4 shows the dynamic responses of the electrical resistance of 90 mol % SnO_2_/10 mol % TiO_2_ and 10 mol % SnO_2_/90 mol % TiO_2_ heterostructures upon interaction with hydrogen at a constant temperature of 400 °C. As one can see in Figure 4a and Figure 4c, the electrical resistance decreases upon admission of reducing gas (hydrogen). Thus we can conclude that globally both heterostructures (SnO_2_-rich and TiO_2_-rich) exhibit n-type conductivity. This is not surprising because usually SnO_2_ and TiO_2_ are treated as n-type semiconductors [32–33]. Moreover, from the comparison of the gas sensing responses given in Figure 4a and Figure 4c it is easily seen that the heterostructure of 90 mol % SnO_2_/10 mol % TiO_2_ is very sensitive even to small H_2_ concentrations (5–300 ppm H_2_, Figure 4a) while the TiO_2_-rich composition, i.e., 10 mol % SnO_2_/90 mol % TiO_2_ requires higher hydrogen concentrations (50–3000 ppm H_2_, Figure 4c).

SEM and HR-TEM images, as well as the results of selected area electron diffraction (SAED) and mapping of elements are given in Figure 4b and Figure 4d for the gas sensing materials given in Figure 4a and Figure 4c, respectively. There are some differences between the microstructure of 90 mol % SnO_2_/10 mol % TiO_2_ and 10 mol % SnO_2_/90 mol % TiO_2_. In the case of TiO_2_-rich heterostructures the grains are larger and spherical (Figure 4d), while for SnO_2_-rich compositions grains are smaller, more irregular in shape and elongated (Figure 4b). The spherical nanograins of TiO_2_ are probably composed of smaller crystallites while separate SnO_2_ grains were not identified by SAED for 10 mol % SnO_2_/90 mol % TiO_2_. Element mapping suggests that a small amount of Sn (Figure 4d) is finely dispersed within the TiO_2_ matrix. In the 90 mol % SnO_2_/10 mol % TiO_2_ heterostructures Ti is well incorporated into SnO_2_ building blocks (Figure 4b).

**Figure 5:**
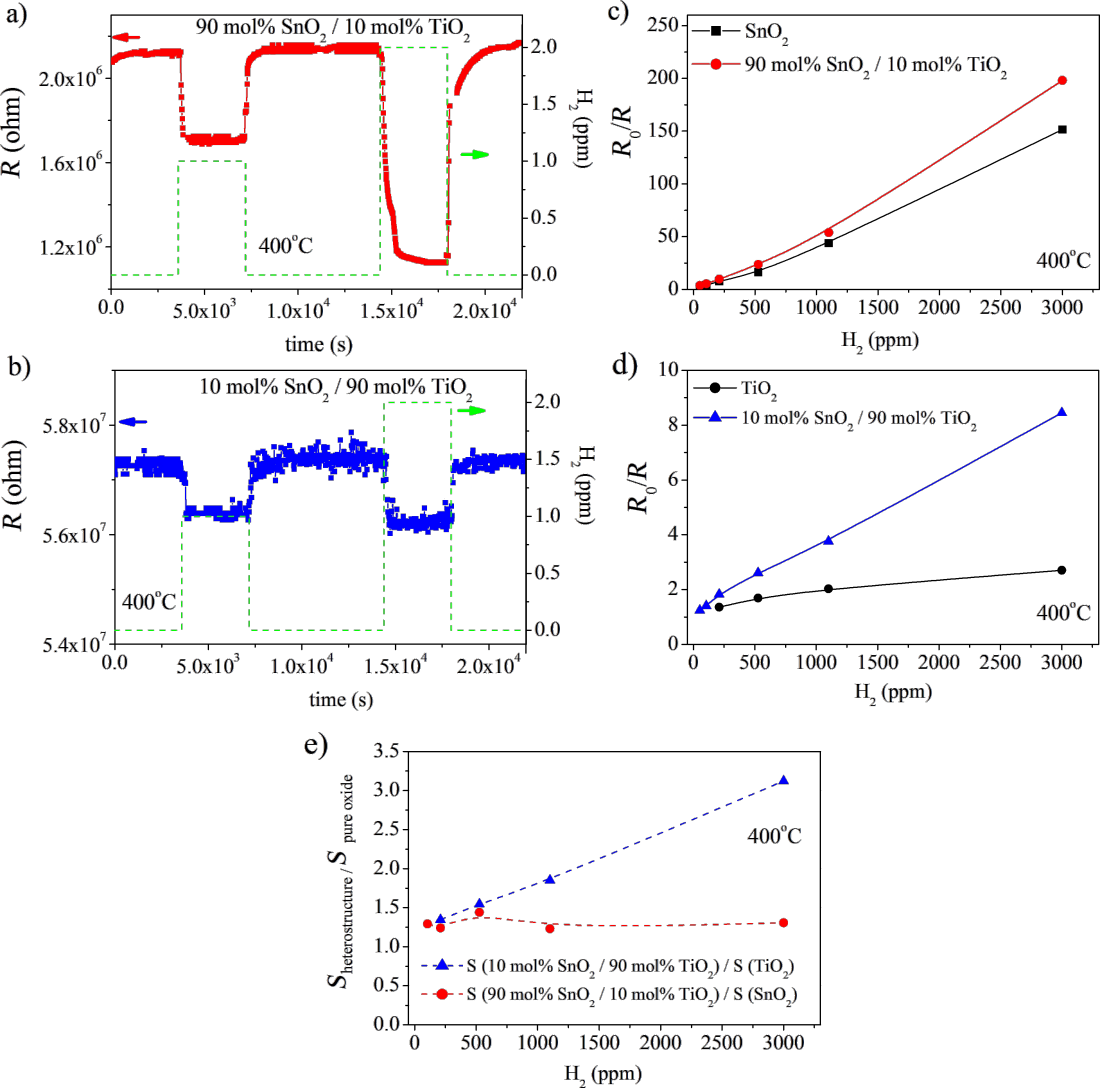
Dynamic changes in the electrical resistance, *R*, of: a) 90 mol % SnO_2_/10 mol % TiO_2_; b) 10 mol % SnO_2_/90 mol % TiO_2_ nanomaterials upon interaction with 1 and 2 ppm of H_2_ along with the corresponding sensor response (*R*_0_/*R*) as a function of H_2_ concentration (c, d). The influence of the formation of heterostructures on the gas-sensing performance is given in e) as the corresponding ratio of responses *S*_heterostructure_ normalized to those of pure SnO_2_ and TiO_2_ (*S*_pure oxide_). The gas-sensor response *S* is defined in Equation 4.

Driven by the promising sensor signal for the step changes in H_2_ concentration (Figure 4), we decided to perform additional measurements in order to determine the hydrogen detection threshold for the studied TiO_2_/SnO_2_ heterostructures. Figure 5a and Figure 5b present dynamic changes in the electrical resistance of 90 mol % SnO_2_/10 mol % TiO_2_ and 10 mol % SnO_2_/90 mol % TiO_2_, respectively, upon interaction with 1 and 2 ppm H_2_. The H_2_ detection threshold for the studied TiO_2_/SnO_2_ heterostructures is lower than 1 ppm, especially in the case of SnO_2_-rich composition (Figure 5a). As one discusses 10 mol % SnO_2_/90 mol % TiO_2_ it appears that at 1 ppm H_2_ the signal-to-noise ratio becomes much worse. However, the sensor signal is still discernible.

Within the studied temperature range SnO_2_-rich nanomaterials exhibit better gas-sensing performance (Figure 5c,d). The larger sensor response, *R*_0_/*R* (by about 20 times) for SnO_2_-rich heterostructures compared to TiO_2_-rich ones is typical as titanium dioxide requires higher temperatures for improved sensing characteristics.

In Figure 5c and Figure 5d one can also analyze the influence of the formation of heterostructures on the sensor response, *R*_0_/*R*. In both cases the sensor response increases compared to the pure oxides. The improvement in gas-sensing by a small addition of TiO_2_ to SnO_2_ was reported previously [5]. The explanation of this phenomenon is based on the charge transfer between TiO_2_ and SnO_2_ due to the differences in the positions of the conduction and valence band edges of both oxides (Figure 1). A similar effect was reported in our previous work for 2 mol % TiO_2_/98 mol % SnO_2_ nanocomposites working as H_2_ sensors [4].

In this work, for the first time, based on Figure 5e, we can conclude that the addition of SnO_2_ to TiO_2_ (10 mol % SnO_2_/90 mol % TiO_2_) has a much more pronounced effect than the addition of TiO_2_ to SnO_2_ (90 mol % SnO_2_/10 mol % TiO_2_). Moreover, one should also take into account the kinetics of interaction described by response and recovery times.

**Figure 6:**
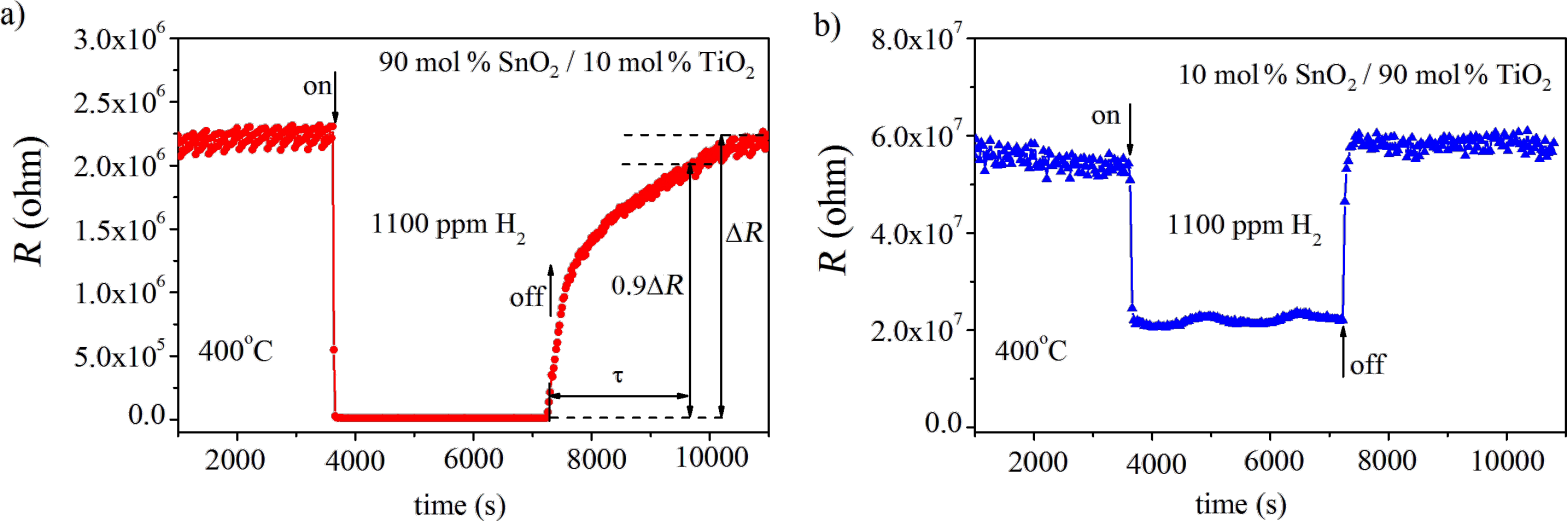
Dynamic changes in the electrical resistance, *R*, of: a) 90 mol % SnO_2_/10 mol % TiO_2_; b) 10 mol % SnO_2_/90 mol % TiO_2_ heterostructures upon interaction with 1100 ppm H_2_. τ denotes the recovery time.

Despite the fact that SnO_2_-rich heterostructures exhibit larger responses to gases over the whole measuring range, it appears that their recovery time, τ, for the sensor to reach 90% of the initial electrical resistance, *R*_0_, is much longer than that of TiO_2_-rich heterostructures at higher H_2_ concentrations (Figure 6). In the case of 90 mol % SnO_2_/10 mol % TiO_2_ (1100 ppm H_2_), τ is about 2500 s, whereas for 10 mol % SnO_2_/90 mol % TiO_2_ (1100 ppm H_2_), τ is less than 30 s. The longer recovery time of SnO_2_-rich sensors can be attributed to a constricted gas desorption that probably results from the differences in the microstructure evidenced by SEM (Figure 4).

A fast desorption process is a prerequisite for the reproducible response and from this point of view TiO_2_-rich heterostructures exhibit better performance at higher H_2_ concentrations (1000–3000 ppm). From the analysis presented in Figure 5 and Figure 6 one can make the conclusion that TiO_2_/SnO_2_ nano-heterostructures can be intentionally modified by changing the chemical composition in order to meet requirements for successful detection of both small (SnO_2_-rich content) and large H_2_ concentrations (TiO_2_-rich compositions).

**Figure 7:**
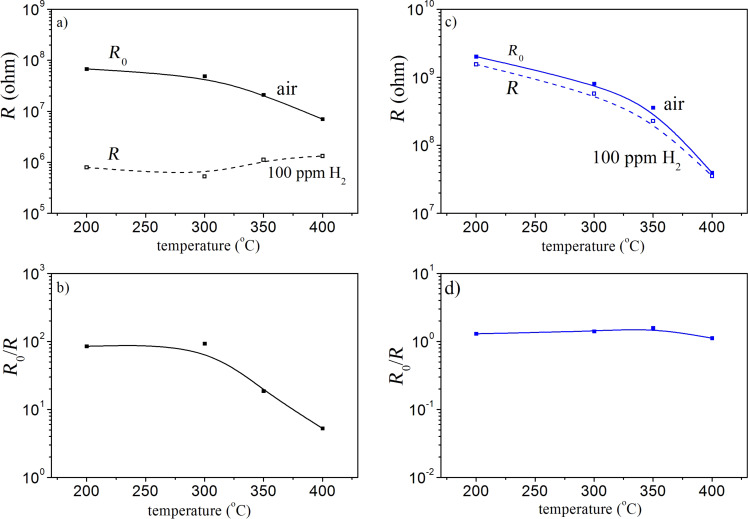
Temperature dependence of the electrical resistance in air, *R*_0_, compared with that upon interaction with 100 ppm H_2_, *R*, together with the sensor response, *R*_0_/*R*, for the samples of 90 mol % SnO_2_/10 mol % TiO_2_ (a, b) and 90 mol % TiO_2_/10 mol % SnO_2_ (c, d).

Figure 7 demonstrates the temperature variation of the electrical resistance in the reference gas (air), *R*_0_, its value upon interaction with 100 ppm H_2_, *R*, as well as the sensor response defined as a ratio of *R*_0_/*R* for the two compositions of 90 mol % SnO_2_/10 mol % TiO_2_ (Figure 7a,b) as well as 10 mol % SnO_2_/90 mol % TiO_2_ (Figure 7c,d).

It can be seen that *R*_0_ decreases with increasing operating temperature. As for *R*, it seems that this effect is more pronounced for the TiO_2_-rich sample. In the case of the SnO_2_-rich composite the electrical resistance *R* upon interaction with 100 ppm H_2_ seems to be independent of the temperature. For 90 mol % SnO_2_/10 mol % TiO_2_, the temperature dependence of *R*_0_/*R* follows *R*_0_ vs temperature, because *R* is almost constant. On the other hand, in the case of the TiO_2_-rich sample both *R*_0_ and *R* exhibit a similar temperature dependence, which leads to a gas response *R*_0_/*R* almost independent of the temperature.

**Figure 8:**
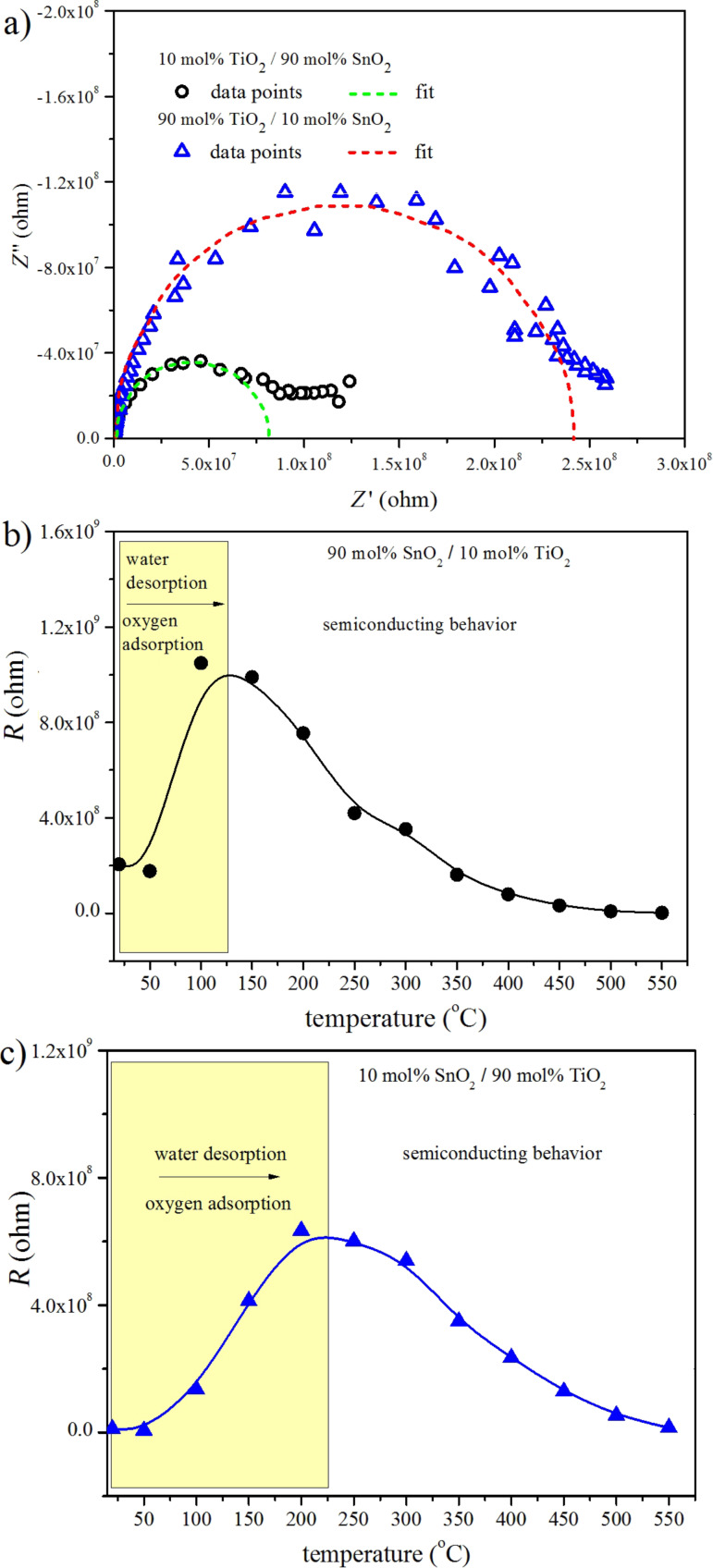
a) Impedance spectra of 90 mol % SnO_2_/10 mol % TiO_2_ and 90 mol % TiO_2_/10 mol % SnO_2_ at 400 °C along with the electrical resistance as a function of the temperature (b, c) obtained on the basis of impedance measurements.

In order to study the electrical properties of TiO_2_/SnO_2_, impedance spectroscopy was applied. Figure 8 presents: a) the impedance spectra obtained at 400 °C as well as the electrical resistance as a function of the temperature for: b) 90 mol % SnO_2_/10 mol % TiO_2_ and c) 10 mol % SnO_2_/90 mol % TiO_2_.

The impedance spectra (IS) in Nyquist representation (Figure 8a) consist of a well-developed semicircle, followed by a deformed semicircle at lower frequencies. The equivalent circuit fitted to all spectra is a loop composed of one resistor *R* in parallel with a constant phase element CPE. Resistor *R* and CPE represent the bulk/surface process and their values have been determined by fitting (Figure 8a). The CPE in the majority of cases resembles a Debye capacitor *C*. The resulting electrical resistance *R* as a function of temperature exhibits a maximum, the position of which *T*_max_ depends on the competing processes water desorption, oxygen adsorption and semiconducting behavior at higher temperatures. As can be seen in Figure 8b, for SnO_2_-rich heterostructures *T*_max_ is about 100–125 °C, while for TiO_2_-rich heterostructures as shown in Figure 8c, *T*_max_ is much higher within the range of 200–250 °C.

From the thermodynamics of chemical reactions it is well known that oxygen adsorption (described, e.g., by the coverage degree Γ) is an exothermic process and decreases with temperature [34]. Under the experimental conditions this situation is given when the adsorption processes remain in thermodynamic equilibrium, i.e., at temperatures larger than a characteristic value *T*_eq_. In the case of oxygen adsorption at the surface of oxides, *T*_eq_ is of the order of 400 °C [35]. At temperatures smaller than *T*_eq_ the coverage degree Γ increases with temperature as described by the laws of chemical kinetics.

The interpretation of the results given in Figure 8, assuming that the resistance changes are related only to the gas adsorption, is based on the fact that the experimental *T*_max_ is much smaller than the theoretically predicted *T*_eq_. At these relatively low temperatures water desorption is believed to predominate over oxygen adsorption. However, both processes are possible. In the literature one can find three types of mechanisms explaining the increase in the surface conductivity in the presence of water vapor as in all these cases the electron concentration is increased [36]. Water adsorption becomes important at temperatures below *T*_max_ and certainly at room temperature.

The subsequent increase in the temperature above 100–200 °C (see Figure 8b,c) leads to a decrease in the electrical resistance, which is a typical effect for semiconductors and is related to the creation of additional charge carriers.

The temperature *T*_max_ at which the semiconducting behavior begins to prevail over water desorption/oxygen adsorption depends on the TiO_2_–SnO_2_ composition. The higher *T*_max_ for TiO_2_-rich heterostructures can be explained on the basis of the higher ionic defect concentration (mainly oxygen vacancies) at the surface of TiO_2_. It is well known that oxygen vacancies act as water adsorption centers. Moreover, in the case of SnO_2_ water adsorption takes place because of the formation of weak van der Waals bonds between water dipoles and lattice ions (Sn^4+^ and O^2−^) [19]. This facilitates water desorption from the surface of SnO_2_-rich heterostructures at lower temperatures.

As one discusses the interaction between the gas phase and the semiconducting sensor, the two-step mechanism described in the Introduction section has to be taken into account. The second step given in a general form by Equation 2 is the surface reduction, which appears upon interaction with hydrogen and can be described in detail as follows [1,37]:

[5]
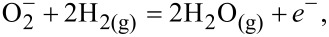


[6]
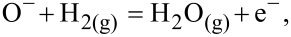


[7]
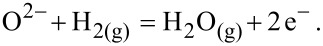


Applying the law of mass action to Equations 5–7 yields:

[8]
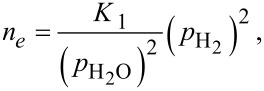


[9]
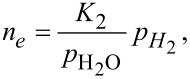


[10]
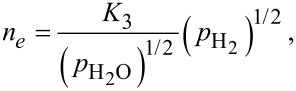


where *n_e_* denotes the concentration of electrons, and *K*_1_, *K*_2_, *K*_3_ are the equilibrium constants of the reactions described by Equations 5–7. The concentration of adsorbed oxygen is assumed to remain constant during its interaction with hydrogen. This is justified by *p*_O2_ >> *p*_H2_ and a high rate of oxygen chemisorption under the experimental conditions [1].

As the electron mobility μ*_e_* is practically independent of the gas partial pressure, and the relationship for the electrical resistivity reduces to ρ = 1/(*e*·μ*_e_·n_e_*) for n-type semiconductors, the 1/*R*(*p*_H2_) dependence assumes the same form as *n_e_*(*p*_H2_) does (Equations 8–10). Thus, *n* = 1/2, 1 or 2 are theoretically predicted for different oxygen species preadsorbed on the surface of the semiconductor.

In the case of formation of oxygen vacancies V_O_, the following reaction could be proposed:

[11]
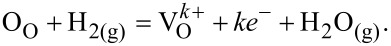


The condition of lattice electroneutrality requires that:

[12]
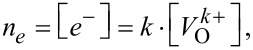


where *k* = 1 or 2 corresponds to singly or doubly ionized defects, respectively.

Applying the law of mass action to Equation 11 (with *k* = 1 or 2) gives power-law coefficients of *n* = 1/2 or 1/3 according to the relation:

[13]
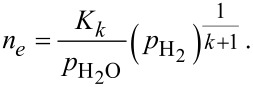


**Figure 9:**
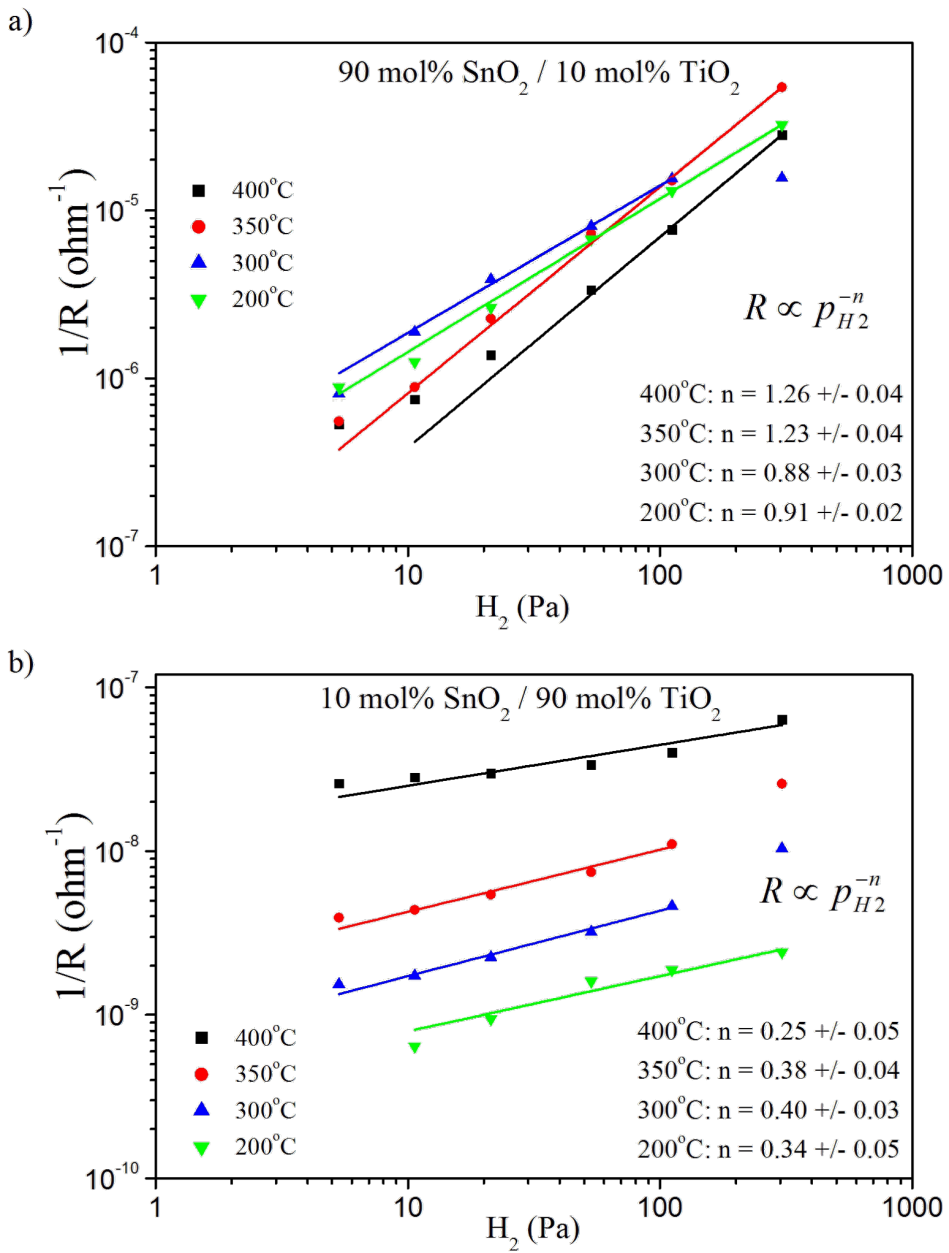
Log–log plot of the inverse of electrical resistance vs the hydrogen partial pressure for: a) 90 mol % SnO_2_/10 mol % TiO_2_ and b) 10 mol % SnO_2_/90 mol % TiO_2_ at temperatures of 200–400 °C.

Figure 9 and Table 4 demonstrate the results of the power-law analysis of the sensor response for: a) 90 mol % SnO_2_/10 mol % TiO_2_ and b) 10 mol % SnO_2_/90 mol % TiO_2_. In the log–log plot the dependence 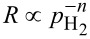 can be fitted with a linear function. The values of the power coefficient *n* corresponding to the predominating form, along with the experimentally determined values (Table 4) can be attributed either to the specific oxygen form preadsorbed at the surface of the sensor or to the oxygen vacancies following the equations given above.

**Table 4:** Theoretical and experimental results of power law analysis.

Theoretically predicted *n*	Equation	Form	Experimentally determined *n*	Figure	Interpretation

2	8	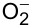	not observed	Figure 9	
1	9	O^−^	0.88–1.26 for 90 mol % SnO_2_/10 mol % TiO_2_	Figure 9a	the ratio of O^−^/O_2_^−^ increases with temperature
1/2	10	O^2−^	not observed for 90 mol % SnO_2_/10 mol % TiO_2_	Figure 9a	
1/2	13	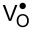	not observed for 10 mol % SnO_2_/90 mol % TiO_2_	Figure 9b	
1/3	13	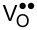	0.25–0.4 for 10 mol % SnO_2_/90 mol % TiO_2_	Figure 9b	our simplified model does not work here, the formation of point defects should be considered

In our case the parameter *n* is around 1 for 90 mol % SnO_2_/10 mol % TiO_2_ at temperatures of 200–300 °C and slightly higher than 1 at 350 and 400 °C (Figure 9, Table 4). According to the literature [19] the adsorbed oxygen forms O_2_^−^, O^−^ and O^2−^ tend to predominate at the surface of SnO_2_ with increasing temperature. Referring to this data one can conclude that in the case of 90 mol % SnO_2_/10 mol % TiO_2_, O_2_^−^ and O^−^ are adsorbed and the ratio of O^−^/O_2_^−^ increases with temperature. Considering the 10 mol % SnO_2_/90 mol % TiO_2_ nanomaterial, *n* is in the range of 0.25–0.40. The reduction of titanium dioxide leads to the formation of oxygen vacancies.

For TiO_2_-rich nanomaterials, the sensing properties cannot be explained within this simplified model. It appears that not only oxygen species preadsorbed on the surface of the semiconductor but also formation of point defects need to be considered.

## Conclusion

1) TiO_2_/SnO_2_ heterostructures are well crystallized, anatase, rutile and cassiterite forms are present. Tin exhibits only the oxidation state 4+.

2) The detection threshold is below 1 ppm H_2_ for SnO_2_-rich heterostructures.

3) The addition of a small amount of SnO_2_ to TiO_2_ has a much more pronounced effect on the sensor response than the modification of SnO_2_ by a small amount of TiO_2_.

4) The recovery time of SnO_2_-based heterostructures is longer than that of TiO_2_-rich samples at higher H_2_ concentrations.

5) TiO_2_/SnO_2_ heterostructures can be intentionally modified in order to meet the requirements for the successful detection of both small (SnO_2_-rich) and large H_2_ concentrations (TiO_2_-rich).

6) The temperature *T*_max_ at which the semiconducting behavior begins to prevail upon water desorption/oxygen adsorption depends on the TiO_2_/SnO_2_ composition.

7) The electrical resistance of the sensor materials exhibits a power-law dependence on the partial pressure of H_2_. In the case of 90 mol % SnO_2_/10 mol % TiO_2_, O_2_^−^ and O^−^ ions are adsorbed and the ratio of O^−^/O_2_^−^ increases with temperature.
